# A new direction for adjunctive therapy of difficult-to-treat depression: examining the role of orexin receptor antagonists

**DOI:** 10.1007/s00406-025-01999-w

**Published:** 2025-05-28

**Authors:** Michael E. Thase

**Affiliations:** Perelman School of Medicine, University of Pennsylvania, Michael J. Crescenz Veterans Affairs Medical Center, 3535 Market Street, Suite 689, Philadelphia, PA 19104 USA

**Keywords:** Major depressive disorder, Antidepressants, Adjunctive therapy, Insomnia, Orexin receptor antagonists, Seltorexant

## Abstract

One of the several pressing unmet needs in the pharmacotherapy of MDD is development of drugs with novel mechanisms of action that can effectively treat depressed patients who do not respond to first- and second-line antidepressants. The value of identifying such a medication would be enhanced if it were also generally well-tolerated and addressed depressive symptoms that are less responsive to SSRIs or SNRIs, such as insomnia or anxiety. This narrative review summarizes the investigation of a novel class of medications originally developed to treat insomnia, the Orexin Receptor Antagonists (ORAs), as adjunctive treatments for depressed patients who have been able to tolerate but who do not obtain an adequate response to standard antidepressants. Although it is likely that the currently approved Dual Orexin Receptor Antagonists (DORAs)—suvorexant, lemborexant and daridorexant—are safe and useful options for concomitant therapy of insomnia in antidepressant-treated patients, these medications have not been approved for this indication. Moreover, DORAs have not been extensively studied as adjunctive therapies for MDD. By contrast, the investigational ORA seltorexant, which is a selective Orexin 2 receptor antagonist, has shown significant antidepressant effects in Phase 2 and Phase 3 trials. Although at least one more unequivocally positive pivotal study will be needed to garner FDA approval for clinical use in the United States, this drug shows promise as a novel and well-tolerated option for patients with difficult to treat depressive episodes.

## Introduction

Although many effective, relatively safe and inexpensive antidepressant medications are now available for treatment of depressive disorders, there are important unmet needs that continue to challenge pharmacotherapists and patients alike. Foremost among these are the need for medications that: (a) have a more rapid onset of action, (b) are helpful for depressed patients who cannot tolerate first- and second-line antidepressants and (c) are effective when several adequate trials of antidepressants have failed to deliver the desired therapeutic effect [37]. Yet another unmet need arises for depressed patients who do obtain some benefit from commonly used antidepressants, but who do not remit fully because of inadequate effect on selected symptoms of their depressive syndrome, such as cognitive symptoms, anxiety or insomnia [22]. Indeed, in the Sequential Treatment Alternatives to Relieve Depression (STAR*D) study, one third of treatment-seeking depressed patients did not remit despite up to four sequential trials of standard treatment options [27]. It is widely presumed that one factor that underpins the unmet needs that compromise the current state of the art of antidepressant pharmacotherapy are a consequence of the field’s 50 + year focus on development of medications that primarily modulate serotonin and/or norepinephrine neurotransmission [37]. This hypothesis is credible because the depressive disorders are a heterogeneous group of illnesses in terms of both clinical features and pathophysiologic correlates [16]. Research designed to identify and test novel targets for pharmacotherapy that are relevant to depressive neurobiology are thus a top priority in the 21 st Century [3, 37].

One such emerging target is the Orexin system [13, 23] and the primary focus of this paper is to review the therapeutic potential of drugs that inhibit Orexin receptors. A brief review the state of the art on the treatment of depressed patients with insomnia will be followed by an examination of the role of the Orexin system in the pathophysiology of depression. The efficacy and safety of currently marketed Orexin Receptor Antagonists for treatment of insomnia are next reviewed, followed by a more detailed summary of the evidence pertaining to the investigational drug seltorexant, which is emerging as one of the leading candidates for inclusion in a new generation of novel antidepressant medications.

## Pharmacotherapy of depression in patients with insomnia

As detailed in reviews written over the past 40 years [11, 14, 36, 38, 40], difficulties with sleep during times of despair—including troubles falling to sleep, light/fitful/broken sleep and waking up hours before sunrise despite only a few hours of sleep—have plagued humans for at least long as our species has been able to record our experiences. As concepts about the conditions that we now call Major Depressive Disorder (MDD) have evolved over the 19th, 20th and 21st Centuries, insomnia symptoms—whether experienced early, middle or late in a night of sleep—have been consistently incorporated in various classifications of depressive states. Moreover, insomnia is not only one of the more commonly experienced symptoms of depressive episodes, but the severity of insomnia symptoms is associated with global episode severity, greater functional impairment and a heightened risk of suicidal behavior [12]. Insomnia is also a risk factor for the onset of a first lifetime episode of depression as well as recurrent depressive episodes and, not uncommonly, insomnia is one of the more persistent symptoms of depressive episodes that do not fully remit with standard therapies. Although insomnia may not be specific to clinical depression, it is an integral aspect of the pathophysiology of depression and can be an important target for antidepressant interventions.

In contemporary practice, most depressed patients treated in North America and Europe are treated with SSRIs or SSRIs, which—while effective for many patients—are not inherently helpful for insomnia symptoms [11]. When insomnia is pronounced or persists despite an overall response to antidepressant therapy, clinicians sometimes opt to prescribe one of the alternative first-line antidepressants, such as mirtazapine or—outside of the US—agomelatine. More commonly clinicians opt to co-prescribe sedative-hypnotic medications or low doses of benzodiazepines such as lorazepam or clonazepam [11, 38, 40]. Because of current efforts in the US to constrain drug costs by limiting access to medications that are still under patent, prescribers usually are not able to use newer generation hypnotic medications of treatment of their patients with MDD. When there are reasons to avoid medications that are classified as controlled substances, many providers opt for concomitant therapy with low doses of trazodone or quetiapine. More recently, low dose formulations of mirtazapine and doxepin also have been introduced for treatment of insomnia. These medications are preferred over sedative hypnotics by many US psychiatrists because they are not habit-forming and could be increased to antidepressant doses if needed to further augment response. When the prescriber wishes to avoid using a controlled substance or the side effects profiles of trazodone or quetiapine, over-the-counter medications such as diphenhydramine and melatonin are used. Although the co-prescription approach to management of depressed patients with insomnia is likely to lessen subjective distress and hasten time to response, the costs and potential side effects concerns resulting from co-prescription should not be underestimated. That said, when considered together, the high prevalence of co-prescription speaks to the unmet need for antidepressant medications that effectively address insomnia.

## The orexin system

Discovered in the late 1990 s, the Orexin system has important regulatory effects on the sleep–wake cycle, appetite, energy regulation, stress response and consummatory or reward-directed behavior [2, 25, 31]. Orexin neurons are localized in the lateral hypothalamus and project to and interact extensively with other neuronal systems implicated in the pathophysiology mood disorders, including those that utilize the classic monoamine neurotransmitters, serotonin, norepinephrine and dopamine, as well as neurons that utilize histamine and acetylcholine [28]. The Orexin system contains two distinct neuropeptides, Orexin-A and Orexin-B, which are secreted by neurons in the lateral hypothalamus. Orexin secreting neurons project widely throughout the brain, where signaling is mediated by two G-protein coupled receptors (OX1R and OX2R). Whereas Orexin-A interacts equally with both types of receptors, Orexin-B is more specific for OX2R [28]. Interestingly, although the Orexin system is found in all vertebrates, only mammals express OX1 receptors [34].

## Orexin receptor antagonists (ORAs) for treatment of insomnia

As the physiological actions of Orexin signaling were being elucidated in the first decade of the 21 st Century, one obvious therapeutic implication was that drugs that could selectively block Orexin receptors could be expected to promote sleep by transiently inhibiting one of the processes that normally promotes wakefulness. Moreover, as relatively selective Orexin Receptor Antagonists (ORAs) began to be identified and were tested in animal models, it was recognized that—unlike the case with conventional sedative-hypnotic compounds, which are either selective or nonselective agonists of gamma aminobutyric acid (GABA) receptors—ORAs could induce sleep without causing excessive sedation or distorting sleep electrophysiology [5, 34, 35]. Moreover, studies using animal models of drug dependence consistently demonstrated that the ORAs’ profiles are indicative of a low risk of addiction [21]. Collectively, these characteristics conveyed several potential advantages for this new class of sleeping medications and might prove particularly useful for people who require chronic treatment for insomnia [5, 19, 21, 34, 35].

**Fig. 1:**
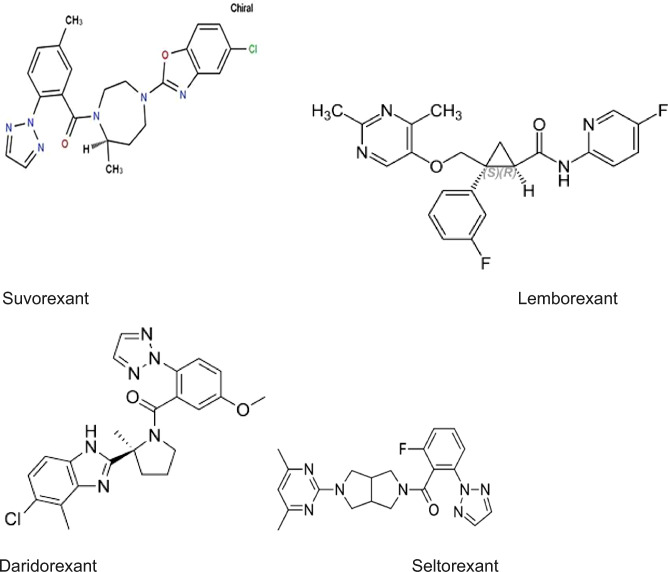
Chemical Structures of Orexin Receptor Antagonists

As briefly summarized below, three members of this new class of new sleeping medications have passed regulatory muster in the United States: suvorexant [15], lemborexant [8] and daridorexant [42] (see Fig. 1). As all three of these medications inhibit both types of Orexin receptors, they are properly grouped together as Dual Orexin Receptor Antagonists (DORAs) [19–21]. Regulatory approval assures that all three of these drugs have shown superiority over placebo in RCTs for acute treatment of insomnia in adults, including those over age 60, with an acceptable profile for safety and tolerability [6, 24]. Although only a small number of studies of DORAs have included active controls (such as zolpidem), results of a network meta-analysis suggest that the benefits and risks of the DORAs are comparable to those of the current first-line sedative-hypnotics [6].

*Suvorexant (Belsomra)*. Approved by the FDA in 2014, suvorexant was the first DORA introduced for treatment of insomnia. Suvorexant is taken 30–60 min before bedtime and has an onset of action of about 30 min; the effective dose range is 5–20 mg. Suvorexant is primarily eliminated by hepatic metabolism and is a substrate of CYP3 A4. As such, it should be prescribed with caution and preferentially at lower doses when treating patients who are taking drugs that inhibit CYP3 A4. Likewise, suvorexant should be prescribed with caution in patients with hepatic impairment.

*Lemborexant (Dayvigo)*. The second DORA approved by the FDA for treatment of insomnia, lemborexant, became available for clinical use in 2020. The effective doses are 5 and 10 mg and the time to peak effect is 1 to 3 h. As compared to suvorexant, lemborexant has a longer elimination half-life (up to 55 h). Lemborexant also is metabolized in the liver and is a substrate of CYP4503 A4 and, as such, similar precautions are necessary.

*Daridorexant (Quviviq)*. Daridorexant was approved by the US FDA for treatment of insomnia in January 2022. Later that year daridorexant became the first DORA to be approved by the EMEA for treatment of insomnia. Daridorexant, which has the shortest elimination half-life (8 h) of the DORA class, has a dose range is 25 to 50 mg, with a time to peak effect of one hour. Like the other DORAs, daridorexant is metabolized in the liver and should be used with caution in patients taking strong CYP4503 A4 inhibitors.

In placebo controlled clinical trials of insomnia, the most common adverse effects of the DORAs are dizziness, nasopharyngitits, headaches, and somnolence. Disturbances of REM sleep are uncommon, but sleep paralysis and nightmares are occasionally reported. The DORAs are contraindicated in narcolepsy.

Unlike conventional sedative-hypnotics, DORAs do not cause respiratory depression and have a lower potential for interactions with alcohol, benzodiazepines and opiates. Despite their classification as controlled substances, they are not associated with tolerance of effects across weeks of treatment and appear to have a low risk of becoming habit forming [9]. As the DORAs do not directly interact with GABAergic neurons, care is needed to minimize the risk of drug withdrawal when cross-titrating with conventional sedative-hypnotics.

At present (circa January 2025), none of the DORAs are commonly prescribed in the US. Despite the strong case that can be made for their broader use, there is one insurmountable fact that limits the current use of DORAs: cost. All three of the DORAs are still under patent protection in the US and such exclusivity means that their cost to patients and insurance companies is substantially greater than the costs of generic formulations of more conventional sedative-hypnotics. It is unlikely that we, as prescribers, will know the true relative value of the DORAs as sedative-hypnotics to our patients until they are available in generic formulations and there are no longer prohibitive differences in cost that inhibit more widespread prescription.

## Orexin and the pathophysiology of depression

Disturbances of sleep, appetite, energy and motivation comprise four of the nine symptom domains for diagnosis of a Major Depressive Episode and, as such, there are compelling reasons to look carefully for dysfunction of the Orexin system in MDD [7, 32, 43]. To date, numerous studies utilizing animal models have been undertaken and, as reviewed elsewhere [7, 13, 23, 43], reduced levels of Orexin have been reported in selected, relevant brain regions in some, but not all studies. In one animal model of depression, mice subjected to several types of acute and chronic stress were shown to have increased expression of Orexin neurons in the hypothalamus and increased OX1R signaling in the amygdala (see [7, 43] for detailed reviews). It seems clear that the experimental manipulations employed in these various studies have a disruptive or dysregulating effect on Orexin signaling, but a causal or mediational relationship has not been definitively established.

In their comprehensive review of the human literature, Fagan, Jones and Baldwin [7] concluded clinical depression is not invariably associated with low levels of Orexin in cerebrospinal fluid, although low Orexin levels may be associated with heightened depressive severity and/or suicidality. Interestingly, as noted earlier, severity of insomnia symptoms also is associated with these two more ominous clinical features [12]. As was the case with the dozens of studies of other implicated neurotransmitters in the cerebrospinal fluid conducted over the decades, inconsistency in results could be a function of sample differences in age, medication status, illness characteristics (e.g., chronicity or symptom severity) or other study characteristics, such as not taking into account potential differences attributable to diurnal variation [29]. It is noteworthy that the only study to assess CSF across the 24-h period found an overall trend toward *elevated* Orexin levels and a significant flattening of the amplitude of diurnal variation [29].

## Studies of ORAs in MDD

*Research with Marketed DORAs*. Given prevalence of insomnia in MDD and the ubiquity of co-prescription of sedative-hypnotic medications during antidepressant treatment, one might assume that the literature would be rich in studies of adjunctive DORA therapy. To the contrary, searches of PubMed and https://clinicaltrials.gov/ reveal a sparse clinical literature and surprisingly few studies (see Appendix 1). In fact, a single small (n = 18) RCT of suvorexant [33] was the only published study identified of any of the approved DORAs conducted in patients with MDD. The FDA’s registry of planned studies of investigational and approved medications, which can be accessed at https://clinicaltrials.gov/ was searched during preparation of this manuscript and there were two planned studies of suvorexant for which no data were reported and no entries of research in MDD for either lemborexant or daridorexant (see Appendix I).

The literature is likewise sparse with respect to standardized case series, case–control studies and other kinds of less methodologically rigorous clinical research. Although the suvorexant was introduced in the United States more than 10 years ago, there is remarkably little clinical data about its effects when used to treatment of insomnia in people suffering from MDD and/or taking antidepressants [7, 10, 32, 43]. In the 18-patient study of Shingetsura et al. [33], patients receiving adjunctive suvorexant had a comparable effect in terms of reduction of insomnia and depression scores as patients treated with adjunctive eszopiclone. Looking at post-marketing experience, inspection of safety data is reassuring and there appears to be little to no risk of exacerbation of depressive symptoms or suicidality following initiation of suvorexant therapy, nor is there much reason for worry about drug-drug interactions with the more commonly prescribed antidepressants [10]. Although clinical experience is necessarily more limited for the more recently introduced DORAs, lemborexant and daridorexant, systematic reviews of safety data reporting to date suggest that the experience using these drugs to treat insomnia in people with a history of MDD and/or taking antidepressant, is similar to that with suvorexant [7, 10].

The only other controlled study of a DORA in MDD in the published literature was, ironically, conducted with an investigational drug—filorexant (MK- 6096)—a drug that is no longer being studied [4]. In this under-powered, double blind proof of concept RCT conducted to determine if filorexant might enhance response to antidepressants, depressed patients were randomly assigned to double blind adjunctive treatment with either filorexant (10 mg at bedtime) or matching placebo. The study’s aims were compromised by slow enrollment and was terminated after 3 years of recruitment having enrolled only 40% of the projected sample size. A total of 128 depressed patients were randomized and, at study endpoint, the difference in Montgomery-Asberg Depression Rating Scale (MADRS) scores was not statistically significant. Although the study’s statistical power was less than planned, a study with 60 subjects per arm is adequate to detect large clinical effects. Yet, the mean difference between the two groups was so small (0.7 points on the MADRS; estimated effect size = Cohen’s *d* < 0.1) that it would likely not have been statistically significant even if the investigators had been able to enroll 300 participants per arm. As such, one can be reasonably confident that—at this dose in this study—the absence of a statistically significant effect was not a Type II error. The filorexant group also did not show a meaningfully greater improvement than the placebo group on the measure of insomnia symptom severity. With respect to tolerability, filorexant was not poorly tolerated and there were no differences in the proportions of participants who either completed the study or withdrew because of adverse events. There was a nominal difference the percentage of participants who experienced at least one adverse event: 42% of the filorexant-treated patients compared to 27% of those in the placebo group. The most common side effect was somnolence, which occurred in 7.8% of the patients taking filorexant and 0% of those taking placebo. Five patients taking active filorexant reported treatment-emergent suicidal ideation compared to one patient taking placebo. Although these differences appear ominous to the eye, they are not a reliable in such a small sample. The clinical development program for filorexant was discontinued in 2015, about one year after suvorexant came to the market, although it is not clear that the manufacturer knew the results of the study when the decision was made.

Much greater research activity has centered on the investigational drug seltorexant, which is a selective ORX2 antagonist [7, 18, 41, 43]. It will be recalled that ORX2 receptors are found in mammals and reptiles alike and, as such, are more evolutionarily conserved than ORX1 receptors. To date, several studies using rodent models of depression have found the seltorexant could prevent or reverse stress-related changes (see Ziemichód et al. [43] for a detailed review) and early studies in depressed humans have yielded promising results suggesting beneficial effects on both depressive symptoms and insomnia [17, 18, 26, 30, 39]. A brief review of the RCTs of seltorexant in MDD patients with insomnia follows.

The first controlled study conducted in patients with MDD [1] used a four-way crossover design to evaluate the impact of a single, double blind bedtime dose of seltorexant (10, 20 or 40 mg) as compared to a matching placebo. Each dosing night was separated by seven days in which participants were unmedicated. This design is novel in that every participant served as his or her own control. The primary dependent measures in this 20-patient trial were total sleep time and sleep efficiency, as derived from the four (post-dose) nights of polysomnography. The investigators found that sleep latency and sleep efficiency improved significantly for all three doses of seltorexant compared to placebo). Visual inspection of results suggested an ascending dose–response relationship with respect to reduction of depressive symptoms. However, the difference between the placebo and 40 mg dose of seltorexant was relatively small and not statistically significant. Overall, results supported the tolerability of seltorexant and suggested that further research may be worthwhile.

In the second study [26] 47 patients with MDD and insomnia were randomized to bedtime treatment with seltorexant 20 mg (*n* = 22), diphenhydramine mg 25 (*n* = 13) or placebo (*n* = 12). This was not an adjunctive therapy study per se and most of the participants (79%) were not taking antidepressants. Nearly three quarters of the participants were women of child-bearing potential, for whom the duration of the double-blind treatment was limited to 10 days because—at that time—there was of a dearth of reproductive toxicology information. Although 16 participants were able to complete 28 days of double-blind treatment period, the small number of was too small to permit a secondary analysis. After 10 days of double-blind treatment, the patients taking seltorexant experienced a nominally greater improvement than those taking diphenhydramine or placebo, although again the differences in symptom scores were modest (1.9 and 1.4 points, respectively, on the Hamilton Depression Rating Scale [HDRS]) and not statistically significant. A post hoc analysis using the 6-item HDRS CORE subscale did reveal a statistically significant difference favoring the seltorexant-treated group over the placebo group, suggesting a more consistent effect was observed after excluding the sleep, appetite and somatization items of the HDRS. Changes in polysomnographic measures were not significantly different between the three groups, providing some initial evidence that this ORA (like the DORAs) does not distort sleep architecture. Although 10 days of treatment with 20 mg of seltrorexant was generally well tolerated, 18% (4/22) of the patients complained of somnolence, compared to none of the patients in the diphenhydramine and placebo groups. Although the data from this pilot study are not overly impressive in retrospect, the study provided the manufacturer enough reason for optimism to move on to Phase 2 clinical studies of seltorexant.

The third published study [30] was a six week, placebo controlled RCT of adjunctive seltorexant therapy, which—at the outset—compared 20 and 40 mg qhs doses in MDD patients who had an inadequate response to antidepressant monotherapy. The study employed an adaptive design, which included a planned interim analysis that focused on the two seltorexant groups. The interim analysis revealed that the larger (40 mg qhs) dose was unlikely to be effective, so that arm was closed and replaced in the randomization scheme by an arm in which patients were treated with a 10 mg qhs dose. At completion of the RCT, a total of 283 patients were randomized to treatment with placebo (n = 137) or 10 mg (n = 33), 20 mg (n = 61) or 40 mg (n = 52) of seltorexant. At study endpoint, there was a significant difference favoring the 20 mg dosing group over the placebo treated group; neither the 10 mg nor the 40 mg dosing groups separated from the group treated with placebo. The advantage observed in the 20 mg seltrorexant groups was 3.1 points on the MADRS, which corresponds to a Cohen’s *d* of approximately 0.30; this is a moderate effect that is comparable to the magnitude of benefit seen in RCTs of other effective adjunctive therapies. Secondary analyses suggested that the antidepressant effect of seltorexant was greater among patients with more severe insomnia symptoms, but not among those with more severe anxiety symptoms. There were no statistically significant differences in side effects between the group treated with 20 mg Seltorexant and the placebo group, although complaints of somnolence were nominally higher among those receiving the active drug (9.8% vs 5.1%). The results of this Phase 2 trial encouraged the manufacturer to advance the drug to the next level of clinical development, with Phase 3 studies focusing on the efficacy and safety of the 20 mg qhs dose as an adjunct to ongoing antidepressant therapy.

Results of a fourth study of seltorexant therapy have not yet been published in the peer reviewed literature but were reported in 2024 as press releases and presented in poster form [39]. This large and well-controlled trial will likely be designated as the first pivotal trial of this compound in the regulatory submission process. This was a study of seltorexant for adjunctive therapy for patients with MDD who had not obtained benefit from one or two adequate trials of standard antidepressants in the current depressive episode. A total of 586 consenting patients were randomized and took at least one bedtime dose of either seltorexant (20 mg; n = 284) or matching placebo (n = 304). Approximately 70% of participants had moderate-to-severed insomnia at baseline. At study endpoint, the patients receiving active seltorexant improved significantly more on the Mongomery Asber Depression Rating Scale than those taking placebo (difference: − 2.6 points; p = 0.007). The difference remained significant on a second, planned analysis that evaluated whether the antidepressant effect was simply an epiphenomenon of improved sleep. After removing the insomnia item from the MADRS total score the mean difference was 2.0 (p = 0.023). Significant benefit was also seen on a third planned analysis of the PROMIS-Sleep Disturbance 8a scale (mean difference 3.7 points, p < 0.001). The 20 mg dose of seltorexant was well-tolerated and there were no significant differences in the incidence of specific side effects.

One additional Phase 3 study of adjunctive seltorexant is nearing completion and a number of other studies have been undertaken (see Appendix I). Among these “non-pivotal” trials, perhaps most noteworthy is a recently published study of seltorexant monotherapy [17]. In this trial of 86 MDD participants randomized to receive 5 weeks of treatment with seltorexant (20 mg or 40 mg) or placebo after not showing a response (i.e., nonresponders experienced less than 30% reduction in HAM-D scores) during an approximately 14 day double blind placebo lead-in. At the end of the active treatment phase, patients treated with 20 mg of seltorexant nightly improved significantly compared to the placebo group whereas those receiving the 40 mg dose of seltorexant did not (mean changes [± SD] were − 7.0 [5.04], − 4.4 [3.67] and − 5.5 [4.34], respectively), seltorexant 40 mg, and − 4.4 (3.67) for placebo (p = 0.0456). Although the advantage of seltorexant in the 20 mg arm over placebo was largely attributable to the subgroup of patients with more severe insomnia symptoms, the treatment benefit remained significant when the contribution of the three sleep items was removed from the HAM-D change scores (nominal p = 0.0289).

At this point, the state of the evidence would suggest that adjunctive seltorexant therapy is well-tolerated at 20 mg/day and it has both antidepressant activity and a beneficial effect on insomnia symptoms when added to ongoing treatment with a conventional antidepressant. It is also true that the weight of the evidence collected to date is not sufficient to warrant review by the FDA. As at least one other larger scale, potentially pivotal randomized controlled study is nearing completion, it is possible that, if statistically significant effects are observed, a New Drug Application may be submitted to the US FDA in late 2025. However, as the failure rate of Phase 3 studies of proven antidepressant treatments has hovered around 50% for the past decade, approval of seltorexant in the near future is hardly a certainty.

## Conclusions

Identification of a medication with a novel mechanism of action that treats depressive episodes characterized by significant insomnia that have not responded to adequate trials of first- or second-line antidepressants would represent an important advance that addresses several unmet needs. Over the past decade, three drugs that share a novel mechanism of action – DORAs – have been introduced for treatment of insomnia and, when compared to compared to commonly prescribed sedative-hypnotics, offer a low risk of addiction/abuse and, when indirectly compared to other, nonhabit-forming options, such as trazodone or quetiapine, a generally more favorable side effect profile. In the decade that has followed the introduction of suvorexant clinical experience indicates that the DORAs can be co-prescribed to treat insomnia in depressed patients taking antidepressants without significant concerns about worsening depression, provoking suicidal behavior or causing drug-drug interactions. However, little evidence has emerged to suggest that the DORAs can be used as adjuncts to first-line antidepressants that have failed to produce an acceptable level of benefit. By contrast, there is a growing body of research that suggests that a novel drug that is highly selective for ORX2 receptors, seltorexant, is safe, reasonably well-tolerated and effective as an adjunct to antidepressants. Although additional research is still needed, seltorexant may well prove to be one of the first members of a new, third generation of novel antidepressants specifically intended for treatment of depressed patients with significant insomnia.

## Appendix I

### Studies of suvorexant

NCT02669030 A Six Week, Randomized, Double-Blind Placebo-Controlled, Suvorexant Augmentation Study of Antidepressant Treatment of Major Depressive Disorder With Residual Insomnia Study “completed” in 2019—no results.

NCT03764683 Suvorexant (Belsomra) for the Treatment of Bipolar Depression With Insomnia. Study withdrawn.

### Studies of seltorexant

NCT04533529 A Study of Seltorexant as Adjunctive Therapy to Antidepressants in Adult and Elderly Participants With Major Depressive Disorder With Insomnia Symptoms Who Have Responded Inadequately to Antidepressant and Long-term Safety Extension Treatment With Seltorexant.

NCT04532749 A Study of Seltorexant as Adjunctive Therapy to Antidepressants in Adult and Elderly Participants With Major Depressive Disorder With Insomnia Symptoms Who Have Responded Inadequately to Antidepressant Therapy.

NCT06559306 Phase 3 Study of Adjunctive Treatment With Seltorexant in Adult and Elderly Participants With Major Depressive Disorder and Insomnia Symptoms.

NCT03321526 A Study to Compare the Efficacy, Safety, and Tolerability of JNJ- 42847922 Versus Quetiapine Extended-Release as Adjunctive Therapy to Antidepressants in Adult Participants With Major Depressive Disorder Who Have Responded Inadequately to Antidepressant Therapy.

NCT03227224 A Study to Evaluate the Efficacy and Safety of JNJ- 42847922 as Adjunctive Therapy to Antidepressants in Adult Participants With Major Depressive Disorder Who Have Responded Inadequately to Antidepressant Therapy.

NCT04513912 A Study of Seltorexant Compared to Quetiapine XR as Adjunctive Therapy to Antidepressants in Adult and Elderly Participants With Major Depressive Disorder With Insomnia Symptoms Who Have Responded Inadequately to Antidepressant Therapy.

NCT04451187 A Study of Oral Seltorexant as an add-on Medication to an Antidepressant on On-road Driving Performance in Participants With Major Depressive Disorder. Study completed and results presented in 2024 as a poster.

NCT04951609 A Study of Seltorexant as Adjunctive Therapy to Antidepressants in Adolescents With Major Depressive Disorder Who Have an Inadequate Response to Selective Serotonin Reuptake Inhibitor (SSRI) and Psychotherapy. Study terminated due to slow enrollment.
